# Frozen section and electron microscopy studies of the infection of the red palm weevil, *Rhynchophorus ferrugineus* (coleoptera:curculionidae) by the entomopathogenic fungus *Metarhizium anisopliae*

**DOI:** 10.1186/s40064-016-3416-6

**Published:** 2016-10-07

**Authors:** Xiaodong Sun, Wei Yan, Jing Zhang, Xiaoqing Niu, Fuheng Li, Weiquan Qin, Guangchang Ma

**Affiliations:** 1 Hainan Key Laboratory of Tropical Oil Crops Biology/Coconuts Research Institute, Chinese Academy of Tropical Agricultural Sciences, Wenchang, 571339 Hainan People’s Republic of China; 2 College of Science, Northeast Agricultural University, Harbin, 150030 Heilongjiang Province People’s Republic of China; 3 Environment and Plant protection Research Institute, Chinese Academy of Tropical Agricultural Sciences, Haikou, 571101 Hainan People’s Republic of China

**Keywords:** *Metarhizium anisopliae*, *Rhynchophorus ferrugineus*, Frozen section, Electron microscopy studies

## Abstract

This study determined the pathogenicity of *Metarhizium anisopliae* strain SD-3 against invasive red palm weevil (RPW), *Rhynchophorus ferrugineus* Olivier (coleoptera:curculionidae) larvae in Hainan Province, China. Inoculation of 1 × 10^8^ conidia/mL caused 100 % mortality of *R. ferrugineus*, indicating that the conidia of strain SD-3 were highly virulent. The process of invasion mechanism was showed by scanning electron microscopy (SEM) and frozen section as follows. Once *R. ferrugineus* was infected by strain SD-3, *M. anisopliae* hyphae first invaded the cuticular and body cavity of *R. ferrugineus*. Secondly, well-developed muscles, fat, tracheaes and digestive tube tissues in the abdomen of *R. ferrugineus* were then decomposed and absorbed by *M. anisopliae* hyphae, leading to the total destruction of the larvae. Finally, *M. anisopliae* hyphae reproduced, resulting in a large number of conidia in the body of RPW. The SEM and frozen section are convenient tools to observe the mode of action of entomopathogenic fungi and to observe how *M. anisopliae* is able to colonize and infect the host.

## Background

Invasive red palm weevil (RPW), *Rhynchophorus ferrugineus* Olivier (coleoptera:curculionidae), is an important pests of a world range of palms of economic importance (Faleiro 2006). *R. ferrugineus* was originally reported in India, while now has been widely distributed in Asia, Africa, Australian (Fiaboe 2012; Kehat 1999; Mankin 2009). In China, *R. ferrugineus* is considered as a quarantine pest, and it has been found in 19 species of 15 palm genera (Dembilio et al. 2009). According to international standards for pest measurements (ISPM). This indicates *R. ferrugineu*s can easily settle down in China, where it potentially poses a great threat to palm trees (Wu et al. 2007).

According to IPM strategy, several control methods have been used to *R. ferrugineu*s invasion of palms, These methods include, cutting down and burning infected palms, trapping adult *R. ferrugineus*, chemical control, host plant resistance, bacteria control, viruses control, nematodes mites control, parasitoid and predator insects, male sterile techniques and so on (Faleiro 2006; Francardi et al. 2013). Beside these, a number of entomopathogenic fungi (*Metarhizium anisopliae, Beauveri bassiana, Aspergillus sp.*, *Trichothecium sp.*, *Penicillium sp.*, *Fusarium sp.*) isolated from naturally infected *R. ferrugineus* as a biological control agent against this weevil (Ghazavi and Avand-Faghih 2002; Gindin et al. 2006; Dembilio et al. 2010; Güerri-Agulló et al. 2011; Francardi et al. 2013), *Metarhizium anisopliae* is one of the most commonly studied species of entomopathogenic fungi, it is environmentally-friendly and harmless to human. However, *M. anisopliae* was discovered in naturally infected *R. ferrugineus* in Egypt and this strain caused a high mortality rate for larval and adult stages only under laboratory conditions (Merghem 2011; Cito et al. 2014).

Despite investigations of infection patterns and histopathology of *M. anisopliae* in selected insects is of economic importance, less study has documented the histopathology of *M. anisopliae* in *R. ferrugineus* (Toledo et al. 2010). Moreover, as the *R. ferrugineus* is highly promiscuous and adults live in aggregation, the fungi could spread in the population, infecting healthy insects by horizontal transmission, as suggested also by Llácer et al. (2013) and Francardi et al. (2013).

Scanning electron microscopy (SEM) has frequently been used to evaluate the infection process of entomopathogenic, Güerri-Agulló et al. (2010) used SEM to study the infection process of *B. bassiana* in *R. ferrugineus* cuticle and whole insects fungi versus their insect hosts. SEM studies by Alcides et al. (2002) provided a valuable insight into the mode of pathogenesis of *M. anisopliae* on western flower thrips. SEM studies of host–pathogen interactions have helped in determining some of the attributes of virulent fungal strains and in the identification of insect barriers to infection (Vestergaard et al. 1999). Frozen section has commonly been used in medical sciences for human pathological analysis due to its fast and stable identification effect (Gal and Cagle 2005), The method used in the preparation of SEM samples should avoid damage to the insect and fungal structures involved in penetration, especially when the objective is to document the infection process (Alcides et al. 2002). The purpose of the present studies, by using Electron Microscopy and frozen section methods, We examined the entire course of infection of *R. ferrugineus* by *M. anisopliae* with particular reference to histopathology of entomopathogenic fungi in *R. ferrugineus*, which could improve the effect of biocontrol and further expand its application in the field.

## Methods

### Fungal isolate and preparation of suspensions

Naturally-dead *R. ferrugineus* cadavers were collected from Wenchang, Hainan Island, China. Samples were soaked in 70 % alcohol for 1 min, and rinsed using sterile distilled water. The cadavers were subsequently surface-sterilized using 0.1 % mercury chloride, followed by three-time rinses in sterile distilled water. Part tissues were cut and inoculated on SDAY containing 40 g/L dextrose, 10 g/L peptone, 10 g/L yeast, 20 g/L agar and 500 µg/mL streptomycin. These tissues were separately placed on sterile petri dishes sealed with preservative film at 28 ± 1 °C, 75 ± 5 % RH for 6 days. Purification was achieved using a monospore culture, named SD-3. Scanning Electron Microscopy (SEM) was performed to study morphologic characteristics of SD-3 (Hitachi S-3000 N) (Driver et al. 2000; Su 2006).

### Experimental insects

A laboratory population of *R. ferrugineus* was established by collecting larvae from infected palm trees in the Wenchang suburb, Hainan, China (Li et al. 2010). Larvae were reared on sugarcane stem tissues at 28 ± 1 °C, 75 ± 5 % RH. After adults emerged, they were placed in jars and supplied with cotton wicks saturated with 8–10 % honey for feeding. Subsequently, eggs were transferred to a moist sterile filter paper within an unsealed Petri dish (12 cm in diameter). Upon hatching, neonate larvae were individually transferred to 50 ml vials containing 10 g weevil’s artificial diet (Martín and Cabello 2006). About 7 days later, laboratory-reared larvae were obtained for further analysis.

### Laboratory bioassays

We selected *R. ferrugineus* larvae of similar size during the feeding period. Conidia of divided purified SD-3 were placed in a sterile 10 mL centrifuge tube containing aqueous 0.1 % Tween 80 and vortexed the mixture for homogenization. Conidial concentration was determined using a hemocytometer. Dilution series of aqueous conidial suspension (1.0 × 10^8^) was prepared after proper modulation, then spayed on larvae. Treated larvae were separately transferred to 50 mL vials containing 10 g weevil’s artificial diet under controlled conditions (28 ± 1 °C, 80 ± 5% R.H). Triplicate was performed for each trial with total 30 insects per process. After the treatment, insects were scored as dead at 24 h interval for 10 days. Meanwhile, dead larvae were selected and tested for potential histopathological changes (Tjandra and Melanie 2011).

### Symptom and histopathological changes of *R. ferrugineus* infected by *M. anisopliae*

Fresh larvae and dead adult were collected in laboratory bioassays avoiding water. Samples were dried via freeze-drying, coated with a gold palladium film, and observed using a Hitachi S-3000 N SEM. The frozen section was carried out on a tissue <1.5 cm wide and 3–5 mm thick. Several drops of optimum-cutting-temperature compound (OCT) were immediately added to the embedding medium on the chuck. The OCT bottom rapidly froze to −28 °C, while the top remained as a clear gel. Larvae were applied to the gel as flat as possible, and a few more drops of OCT were added to complete freeze the specimen in less than one min. After embedding, frozen blocks were cut into 3–5 μm sections at −22 °C and stained as described below: (a) immersion in AFF fixative (glacial acetic acid: alcohol = 1:1) for >30 s; (b) washing with distilled water for ca. 10 s; (c) immersion in hematoxylin for 2 min; (d) immersion in 0.1 % ammonium hydroxide solution for 30 s; (e) washing in running tap water for 30 s; (f) immersion in 80 % alcohol for 2 min; (g) immersion in eosin for 1 min; Histological sections were dehydrated using serial alcohol solutions (80, 95, 95, and 100 % alcohol and xylene for 3 times), mounted in permount, and observed and photographed using SEM (Dong et al. 2009; Toledo et al. 2010).

## Results

### Fungal morphology of fungi

The morphology of strain SD-3 was typical to the genus *M. anisopliae*. It grew fast on SDAY and PDA plates. Round colonies on agar plates were white or cream, with villous and the divergence of hollow hyphae on the surface. Punctiform olive green formed in the middle of the colony, which turned darker during growth and resulted in tawny color at the back of plates.

**Fig. 1:**
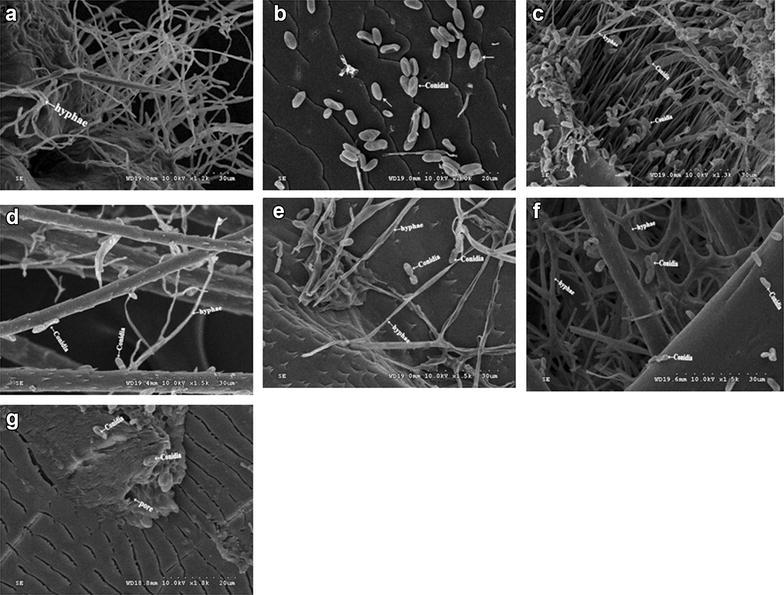
Scanning electron microscope micrographs of *Metarhizium anisopliae* SD-3 on *R. ferrugineus.* **a** *M. anisopliae* SD-3 hyphae (*arrows*) enclosed with the hair situated on the cuticle; **b** *M. anisopliae* conidia in the second antennal segment (*arrows*);** c** *M. anisopliae* conidia and hyphae in the antennal sensory hairs; **d** *M. anisopliae* conidia and hyphae (*arrows*) grow on the hairs of the abdomen; **e** *M. anisopliae* germ tube penetrating through the cuticle of the abdomen with the adult female; **f** *M. anisopliae* conidia and hyphae (*arrows*) enclosed within the cuticle and hairs of the tibia; and **g** *M. anisopliae* conidia (*arrows*) near to the spiracle

The morphology of hyphae and conidial of *M. anisopliae* SD-3 from *R. ferrugineus* cadavers were showed in Fig. 1. The mycelium was smooth and hyaline, well-branched and separated, with major hyphae up to 2.0–3.2 μm wide. Conidiophores were podgy, simple or highly branched, with 2–5 sterigma at the branch top. Conidia were colorless, elliptic and cylindrical, with obtuse ends. Most conidia were 4.8–5.5 μm × 2.0–2.5 μm in length. Growth temperatures were 28 °C.

### Laboratory bioassays

The mortality rate positively increased with conidial concentration (P < 0.05). Conidia were found highly virulent to *R. ferrugineus* and caused approximately 100 % mortality 8 days post-inoculation of 1 × 10^8^ Conidia/mL conidia. To the contrary, control group showed significantly lower mortality rates (3.33 ± 2.87 %)

### Symptom and histopathological changes

SEM micrographs for cuticle and appendages of adult females were showed in Fig. 1b–g. Entomopathogenic fungi predominantly invaded *R. ferrugineus* through the external cuticle (Fig. 1a, e). When *R. ferrugineus* was treated with *M. anisopliae* SD-3, fungal propagules adhered to the cuticular surface and fungal conidia germinated, until appressoria were developed to start the penetration stage at third day (Fig. 1f). Hydrophobic conidia of SD-3 strain were found attached to all of the adult body, with a preference to surfaces containing hairs (Fig. 1c–e). SD-3 conidia were concentrated nearby and enclosed within the pores (Fig. 1g).

**Fig. 2:**
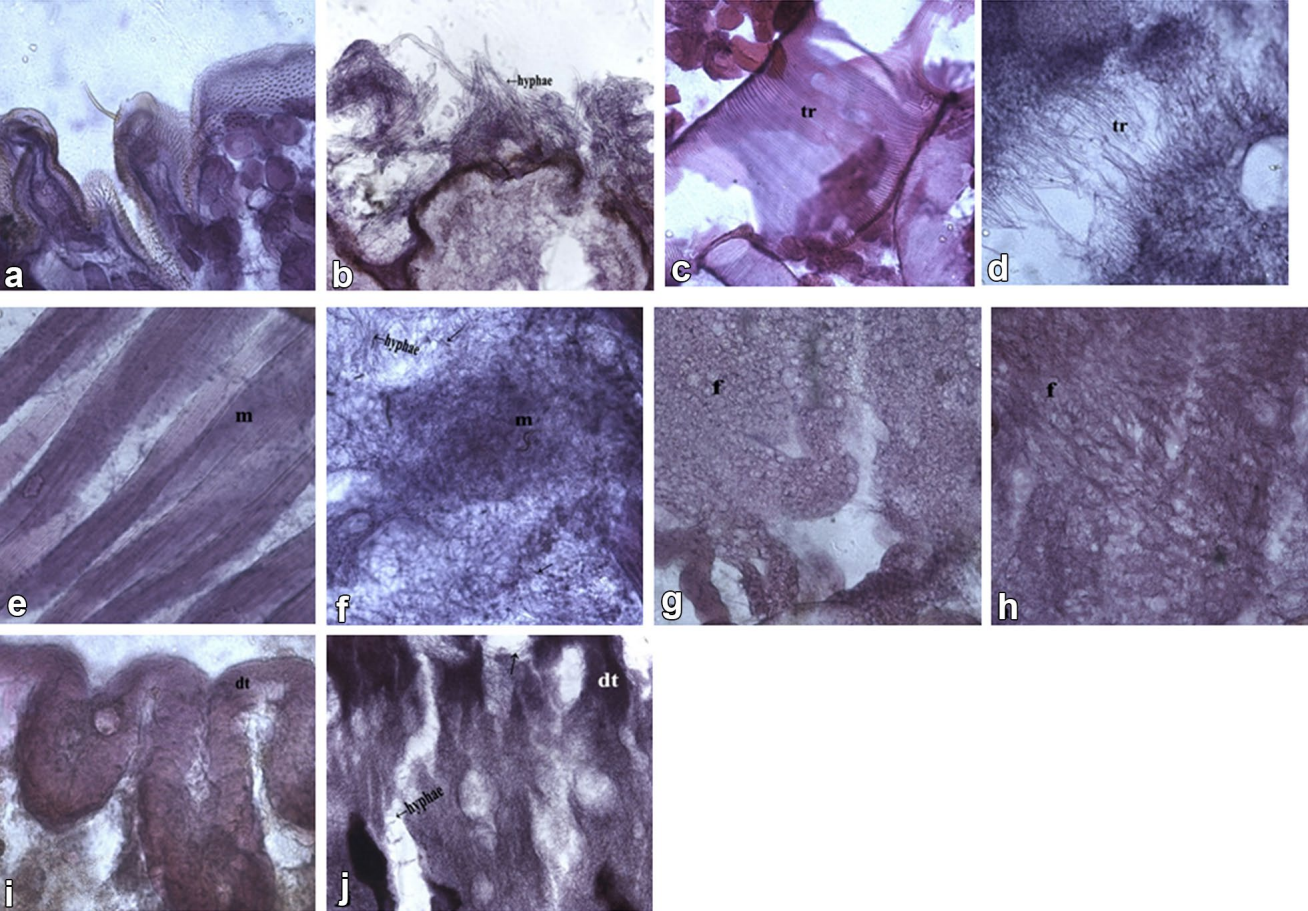
Sagittal sections of *R. ferrugienus* infected by *Metarhizium anisopliae* SD-3. **a** Healthy cuticle; **b** *M. anisopliae* hyphae (*arrows*) penetrating through the cuticle of intersegmental membrane; **c** healthy trachea; **d** *M. anisopliae* hyphae (*arrows*) near the tracheae (tr), Taenidium of infected larvae slowly disappeared at 72 hpi; **e** healthy muscle tissue (m); **f** muscle tissue (m) were decomposed to the net structure by the hyphae (*arrow*) of *M. anisopliae* at 96 hpi; **g** healthy fat tissue; **h** fat tissues (f) were decomposed to the net structure by the hyphae (*arrows*) of *M. anisopliae* at 72 hpi. **i** Healthy digestive tube (midgut) (dt); **j** digestive tube (dt) was decomposed and absorbed by the hyphae (*arrows*) of *M. anisopliae* at 96 hpi

Results of light microscopy for larvae healthy and pathologic tissue were shown in Fig. 2a–j. Cuticle-degrading enzymes such as proteases, chitinases, and collagenases dissolved the cuticle, thus hyphae grew and branched while invading the integument and body cavity of the *R. ferrugineus* (Fig. 2b). *M. anisopliae* SD-3 conidia were found trapped and tightly bound to these hairs (Fig. 2h). Hyphae secreted different metabolic enzymes and destruxins. Well-developed muscles, fat and tracheaes tissue (Fig. 2c, e, g) in the *R. ferrugineus* abdomen were decomposed and absorbed by hyphae, further formed the net structure (Fig. 2d, f, h).After entering the body cavity, hyphae seriously destroyed hemolymph, various tissues and pipelines. Digestive tube (midgut) was decomposed and absorbed by hyphae (Fig. 2j). Finally, hyphae reproduced and resulted in a large number of conidia in the *R. ferrugineus* body. Thus, the whole inner structure of *R. ferrugineus* was destroyed by *M. anisopliae* SD-3 hyphae.

## Discussion

This study showed entomopathogenic fungus *M. anisopliae* SD-3 was highly virulent to *R. ferrugineus*, a serious pest of various palm species. Under the premise of harmless effect to environment or non-target organisms, biological control with pathogenic fungi would offer long-term insect control (Khetan 2001). The pathogenic fungi seem like to survival and distribute with the *R. ferrugineus* in its dark and humid surroundings. *M. anisopliae* is applied as conidia or mycelia in various formulations. By way of making the insects infected through the induction of a fungal epizootic, new conidia and viable cells are produced to spread to the health insects and thus achieve the control effect (Genthner et al. 1997). After the series of adhesion, prepenetration growth, penetration into the host, and settling down of the pathogen in the host, the insects would be infected by *M. anisopliae* (Lattanzio et al. 2006).

Most entomopathogenic fungi enter in the host by penetrating through the host cuticle. In the course of fungal infection, the fungi are adsorbed on the host cuticle in the first step before penetration (Urquiza and Keyhani 2013). Dong et al. (2009) proposed adhesion to occur at three successive stages: (1)adsorption of the fungi propagules to the cuticular surface; (2)adhesion or consolidation of the interface between pre-germinant propagules and the epicuticle; (3)fungi germination and development at the insect cuticular surface, until appressoria are developed to start the penetration stage. Infection will proceed after a successful penetration being achieved. In addition, we found intersegmental membrane and hair was the first spot invaded by *M. anisopliae* when *R. ferrugineus* larvae were infected by this entomopathogenic fungus (Fig. 1). This could be attributed to relatively thin chitin layers in the intersegmental membrane, which favored infection by germ tubes. During the cultivation of dead larvae on moist sterile filter paper, white hypha first occurred at the intersegmental membrane and hair associated with most abundant conidia, as well as longest germ tubes and hyphae. In addition, germination of conidia was first observed in the intersegmental membrane. Together these observations indicated that intersegmental membrane and hair was the weak point subject to *M. anisopliae* infection.

Histological section showed that before *R. ferrugineus* larvae died due to infection, associated body tissues were affected to different degrees, leading to obvious lesions. Direct death reason of the host remained unclear. It was likely that larva body tissues were seriously damaged due to infection by *M. anisopliae*, preventing normal larva physiological activities. Along with mycelia growth, larva bodies (muscles, fat, tracheaes digestive tube) were occupied by a large amount of fungal hypha (Fig. 2), which exhausted nutrients and impeded fluid circulation, leading to physical starvation and metabolic disorders. Thus, hypha invasion could cause body tissue failure of larva, which died due to the incapability of normal physiological activities.

Another reason caused larva death might be physiological and biochemical changes in infected larva body. Sloman and Reynolds (1993) suggested mycotoxin as the true reason for the death of insect infected by many imperfect fungi. Such toxin not only inhibits the immune function of host, but also affects associated central nervous system and has partial pathogenic effect, thus promoting the death of host. However, Wang and You (1999) indicated the uncertainty in functional mechanism of such toxin during relevant infection process. These authors only found pure toxin caused immune pressure, muscle paralysis and malpighian tubule damage in the host. Further study is needed to demonstrate whether mycotoxin produced by *M. anisopliae* was the primary reason caused the death of *R. ferrugineus*.

We used SEM and frozen section to observe the fungal infection. We also first applied frozen section to study histopathological mechanisms of *M. anisopliae* SD-3, and developed a set of methods suitable for studying high-fat larvae. In contrast, research of insect pathology has mainly employed traditional paraffin section, which usually takes 3–5 days for sample preparation and pre-treatment. By comparison, frozen section only requires 30–60 min for sample processes. This contributed to experiment effectiveness and reduced impacts of prolonged fixation on samples.

When collecting fresh samples of histological sections, caution must be taken to avoid water. Temperature is the most important factor for frozen section. As larvae contain substantial fat, high temperature may cause adhesive due to incomplete freeze. On the other side, low temperature may increase the fragility of samples which are easy to break during sectioning. Based on our observations in this work, we suggest rapid freeze at −28 ± 1 °C and section at −22 ± 1 °C yield optimum results.

## Conclusions

In summary, this study showed high susceptibility of *R. ferrugineus* larvae to certain concentrations of local *M. anisopliae* SD-3. We further demonstrated the histopathological mechanism of this entomopathogenic fungus in *R. ferrugineus*. This study allowed the observation of the different phases of the disease cycle, and further demonstrated the importance of understanding these phases in selecting isolates for biological control of *R. ferrugineus*.

## Abbreviations

*M. anisopliae*: *Metarhizium anisopliae*
*B. abassiana*: *Beauveri abassiana*
RPW: red palm weevil
*R. ferrugineus*: *Rhynchophorus ferrugineus* Olivier (coleoptera:curculionidae)

## References

[CR1] Alcides MJ, Sérgio BA, Rogério BL, Pedro MOJN, Roberto MP, Solange AV (2002). External development of the entomopathogenic fungi *Beauveria bassiana* and *Metarhizium anisopliae* in the subterranean termite Heterotermes tenuis. Sci Agr.

[CR2] Cito A, Mazza G, Strangi A, Benvenuti C, Barzanti GP, Dreassi E, Turchetti T, Francardi V, Roversi PF (2014). Characterization and comparison of *Metarhizium* strains isolated from *Rhynchophorus ferrugineus*. FEMS Microbiol Lett.

[CR3] Dembilio Ó, Jacas JA, Llácer E (2009). Are the palms *Washingtonia filifera* and *Chamaerops humilis* suitable hosts for the red palm weevil, *Rhynchophorus ferrugineus* (coleoptera:curculionidae). J Appl Entomol.

[CR4] Dembilio Ó, Quesada-Moraga E, Santiago-Álvarez C, Jacas JA (2010). Potential of an indigenous strain of the entomopathogenic fungus *Beauveria bassiana* as a biological control agent against the Red Palm Weevil, *Rhynchophorus ferrugineus*. J Invertebr Pathol.

[CR5] Dong CJ, Zhang JM, Huang H, Chen WG, Hu YY (2009). Pathogenicity of a new China variety of *Metarhizium anisopliae (M. Anisopliae var. Dcjhyium)*to subterranean termite *Odontotermes formosanus*. Microbiol Res.

[CR6] Driver F, Milner RJ, Trueman JWH (2000) A taxonomic revision of *Metarhizium* based on a phylogenetic analysis of rDNA sequence data. Mycol Res 104:134–150. http://cordyceps.us/files/Driver_etal_2000.pdf

[CR7] Faleiro JR (2006). A review of the issues and management of the red palm weevil *Rhynchophorus ferrugineus* (coleoptera: rhynchophoridae) in coconut and date palm during the last one hundred years. Int J Trop Insect Sci.

[CR8] Fiaboe KKM, Roda AL (2012). Predicting the potential worldwide distribution of the Red Palm Weevil *Rhynchophorus ferrugineus* (Olivier) (coleoptera:curculionidae) using ecological niche modeling. Fla Entomol.

[CR9] Francardi V, Benvenuti C, Barzanti GP, Roversi PF (2013). Autocontamination trap with entomopathogenic fungi: a possible strategy in the control of *Rhynchophorus ferrugineus* (Olivier) (coleoptera:curculionidae). Redia.

[CR10] Gal AA, Cagle PT (2005). The 100 year anniversary of the description of the frozen section procedure. J Am Med Associ.

[CR11] Genthner FJ, Foss SS, Glast PS (1997). Viruence of *Metarhizium anisopliae* to embryos of the grass shrimp *Palaemonetespugio*. J Invertebr Pathol.

[CR12] Ghazavi M, Avand-Faghih A (2002). Isolation of two entomopathogenic fungi on red palm weevil *Rhynchophorus ferrugineus* (Oliv.) (coleoptera:curculionidae) in Iran. Appl Entomol Phytopathol.

[CR13] Gindin G, Levski S, Glazer I, Soroker V (2006). Evaluation of the entomopathogenic fungi *Metarhizium anisopliae* and *Beauveria bassiana* against the red palm weevil *Rhynchophorus ferrugineus*. Phytoparasitica.

[CR14] Güerri-Agulló B, Gómez-Vidal S, Asensio L, Barranco P, Lopez-Llorca LV (2010). Infection of the red palm weevil (*Rhynchophorus ferrugineus*) by the entomopathogenic fungus *Beauveria bassiana*: a SEM study. Microsc Res Tec.

[CR15] Güerri-Agulló B, López-Follana R, Asensio L, Barranco P, Lopez-Llorca LV (2011). Use of a solid formulation of *Beauveria bassiana* for biocontrol of the red palm weevil (*Rhynchophorus ferrugineus*) (coleoptera:dryophthoridae) under field conditions in SE Spain*. Fla Entomol.

[CR16] Kehat M (1999). Threat to date palms in Israel, Jordan and the Palestinian authority by the red palm weevil, *Rhynchophorus ferrugineus*. Phytoparasitica.

[CR17] Khetan SK (2001). Microbial pest control New York: marcel Dekker. J Phytopathol.

[CR18] Lattanzio V, Lattanzio VMT, Cardinali A (2006). Role of polyphenols in the resistance mechanisms of plants against fungal pathogens and insects. Phytochemistry.

[CR19] Li L, Qin WQ, Ma ZL (2010). Effect of temperature on the population growth of *Rhynchophorus ferrugineus* (coleoptera:curculionidae) on sugarcane. Entomol Soc Am.

[CR20] Llácer E, Santiago ÁC, Jacas JA (2013). Could sterile males be used to vector a microbiological control agent? The case of *Rhynchophorus ferrugineus* and *Beauveria bassiana*. Bull Entomol Res.

[CR21] Mankin RW, Samson PR, Chandler KJ (2009). Acoustic detection of Melolonthine larvae in Australian Sugarcane. J Econ Entomol.

[CR22] Martín MM, Cabello T (2006). Manejo de la cría del picudo rojo de la palmera, *Rhynchophorus ferrugineus* (Olivier, 1790) (coleoptera:dryophthoridae), en dieta artificial y efectos en su biometría y biología. Bol San Veg Plagas.

[CR23] Merghem A (2011). Susceptibility of the red palm weevil, *R. ferrugineus* (Olivier) to the green muscardine fungus *Metarhizium anisopliae* (metsch.) in the laboratory and in palm tree orchards Egypt. J Biol Pest Control.

[CR24] Sloman IS, Reynolds SE (1993). Inhibition of ecdysteroid secretion from Manduca prothoracic glands in vitro by destruxins—cyclic depsipeptide toxins from the insect pathogenic fungus *Metarhizium anisopliae*. Insect Biochem Molec Biol.

[CR25] Su XQ (2006). A new species of *Pythium* isolated from mosquito larvae and its ITS region of rDNA. Mycosystema.

[CR26] Tjandra A, Melanie REP (2011). Cellular and humoral immune defenses of *Oxya japonica* (orthoptera:acrididae) to entomopathogenic fungi *Metarhizium anisopliae*. Entomol Res.

[CR27] Toledo AV, de RemesLenicov AMM, LópezLastra CC (2010). Histopathology caused by the entomopathogenic fungi, *Beauveria bassiana* and *Metarhizium anisopliae*, in the adult planthopper, *Peregrinus maidis*, a maize virus vector. J Insect Sci.

[CR28] Urquiza AO, Keyhani NO (2013). Action on the surface: entomopathogenic fungi versus the Insect Cuticle. Insects.

[CR29] Vestergaard S, Butt TM, Bresciani J, Gillespie AT, Eilenberg J (1999). Light and electron microscopy studies of the infection of the western flower thrips *Frankliniella occidentalis* (thysanoptera:thripidae) by the entomopathogenic fungus *Metarhizium anisopliae*. J Invertebr Pathol.

[CR30] Wang HC, You MS (1999). *Metarhizium anisopliae* to insect invasion mechanism. Microbiol.

[CR31] Wu GC, Luo XY, Heng H, Dong YX, Ye LH (2007). Risk analysis of alien invasive pest *Rhynchophorus ferrugineus* (Olivier). China forestry Sci Tec.

